# Frequency-Dependent Changes of Local Resting Oscillations in Sleep-Deprived Brain

**DOI:** 10.1371/journal.pone.0120323

**Published:** 2015-03-23

**Authors:** Lei Gao, Lijun Bai, Yuchen Zhang, Xi-jian Dai, Rana Netra, Youjiang Min, Fuqing Zhou, Chen Niu, Wanghuan Dun, Honghan Gong, Ming Zhang

**Affiliations:** 1 Department of Medical Imaging, the First Affiliated Hospital of Xi’an Jiaotong University, Xi’an, China; 2 Department of Radiology, the First Affiliated Hospital of Nanchang University, Nanchang, Jiangxi Province, China; 3 The Key Laboratory of Biomedical Information Engineering, Ministry of Education, Department of Biomedical Engineering, School of Life Science and Technology, Xi’an Jiaotong University, Xi’an, China; 4 Zonglian Experimental Class, Xi’an Jiaotong University, Xi’an, China; 5 Acupuncture & Rehabilitation Department, Affiliated Hospital of Jiangxi University of Traditional Chinese Medicine, Nanchang, Jiangxi Province, China; University of Florida, UNITED STATES

## Abstract

Sleep deprivation (SD) adversely affects brain function and is accompanied by frequency dependent changes in EEG. Recent studies have suggested that BOLD fluctuations pertain to a spatiotemporal organization with different frequencies. The present study aimed to investigate the frequency-dependent SD-related brain oscillatory activity by using the amplitude of low-frequency fluctuation (ALFF) analysis. The ALFF changes were measured across different frequencies (Slow-4: 0.027–0.073 Hz; Slow-5: 0.01–0.027 Hz; and Typical band: 0.01–0.08 Hz) in 24 h SD as compared to rested wakeful during resting-state fMRI. Sixteen volunteers underwent two fMRI sessions, once during rested wakefulness and once after 24 h of SD. SD showed prominently decreased ALFF in the right inferior parietal lobule (IPL), bilateral orbitofrontal cortex (OFC) and dorsolateral prefrontal cortex (DLPFC), while increased ALFF in the visual cortex, left sensorimotor cortex and fusiform gyrus. Across the Slow-4 and Slow-5, results differed significantly in the OFC, DLPFC, thalamus and caudate in comparison to typical frequency band; and Slow-4 showed greater differences. In addition, negative correlations of behavior performance and ALFF patterns were found mainly in the right IPL across the typical frequency band. These observations provided novel insights about the physiological responses of SD, identified how it disturbs the brain rhythms, and linked SD with frequency-dependent alterations in amplitude patterns.

## Introduction

Sleep is supposed to be beneficial to synaptic renormalization that is sustainable and ensures homeostatic changes in brain’s symphony [1]. Sleep deprivation (SD) has been associated with deteriorative attention, memory, decision making and executive function [2,3,4,5,6,7,8,9,10,11]. In this regards, sleep deprivation may stand for an increasing burden on integrity of brain’s functional architecture. Resting-state fMRI offers a suitable avenue to characterize the brain intrinsic functional architecture associated with sleep deprivation from low-frequency (0.01–0.08 Hz) blood oxygen level dependent (BOLD) signal dynamics.

Resting state fMRI studies have found altered functional connectivity in the sleep-deprived brain [12,13,14,15,16,17]. Evidence for the impact of sleep deprivation on the default mode network (DMN) [18,19] has shown significantly disrupted patterns of task-related deactivation, resulting in a double dissociation within anterior as well as posterior midline regions of the DMN [12]. Besides, multiple observations of altered connectivity intra- and inter- various resting-state networks have been reported for SD [12,13,14,15,16,20,21,22]. Such findings suggest that SD disturbs ongoing patterns of resting-state activity for internal processing of information.

Recent studies have demonstrated that the oscillatory dynamics of BOLD signal is sensitive to specific frequency bands [23,24,25]. For example, Zuo et al. [24] have showed that amplitude of low-frequency fluctuations (ALFF) in the slow-4 band (0.027–0.073 Hz) were higher than that of in the slow-5 (0.01–0.027 Hz) in a wide range of brain regions such as the basal ganglia, thalamus, and precuneus (PCu). Other studies indicate that different frequency bands can distinguish alterations in various diseases such as schizophrenia, chronic back pain, mild cognitive impairment (MCI) & Alzheimer's disease (AD), and personality traits [26,27,28,29,30]. However, previous studies have demonstrated that sleep deprivation induces frequency specific changes in the EEG during wakefulness [31,32,33]. It would be noted that brain rhythms of intrinsic BOLD fluctuations represent an organized architecture of brain function and may be altered in sleep-deprived brain. SD means a burden to restore the oscillatory configuration that is optimal for information processing. Therefore, examining changes in the brain’s BOLD oscillatory dynamics may provide us with novel insight regarding to the physiological responses underlying SD.

In this study, we examine changes in ALFF across different frequencies (Slow-4: 0.027–0.073 Hz; Slow-5: 0.01–0.027 Hz, and typical band: 0.01–0.08 Hz). ALFF needs no priori and offers a valuable tool to investigate local features of brain oscillatory activity [34,35,36]. We hypothesized that the local resting brain activity would be altered after 24h of sleep deprivation and the alterations may be frequency-dependent.

## Materials and Methods

### Subjects

Sixteen healthy volunteers (8 females, 8 males) with a mean age of 22.1 ± 0.8 years were recruited by campus advertising, with the approval by the medical research ethics committee and institutional review board of the First Affiliated Hospital of Nanchang University. The written informed consent for participation was obtained. Participants had an average of 15.7 ± 1.2 years of education. All of them were right handed (as determined by the modified Edinburgh Handedness Questionnaire) and had negative history of neurological, psychiatric, or sleep disorders. There were no history of drug abuse and current use of anti-depressant or hypnotic medications.

### Sleep deprivation and experimental protocol

Subjects were scheduled for fMRI measurements starting at 7:00 PM. There are one fMRI scanning following the individual’s habitual sleep schedule called a rested wakeful night (RW) and the other fMRI scanning after a night of total SD. The sequence of experiments was counterbalanced across sessions and approximately two weeks apart between two experiments. Subjects were forbidden to tea, coffee or caffeine content drinks and alcohol intake for 72 h before and during the fMRI examination. Sleep logs were kept from a week prior to the study night. During sleep deprivation, subjects stayed up the whole night in the laboratory under the direct supervision of a researcher.

Participants showed normal sleep quality as assessed using the Pittsburgh Sleep Quality Index (PSQI) [37] (mean±SD, 1.5±0.97) and normal daytime sleepiness as assessed using the Epworth Sleepiness Scale (ESS)[38] (mean±SD, 6.44±2.07). They had a BMI (in kg/m^2^) of 17.5–22, and were free of nightshift work. Before experiment (approximately 4 weeks), subjects were required to sleep 7–9 h/night, preceding 01:00 a.m on average and keep sleep logs.

A behavior test was performed by each subject prior to fMRI scanning. Word stimuli were displayed in the center of 15.4 inch color monitor on an ASUS F5SL notebook PC running DMDX v.3.0.4 [39]. Screen resolution was set at 800×600 pixel, 16 bit (65,536 colors). White words of 10 mm in size were presented on a black background. During the experiment, each pair of stimuli was presented for 900 ms separated by a blank screen for 500 ms. Subjects had 2,500 ms between trials to judge whether the two words were semantically related or not. A positive response was indicated by pressing the right button using the middle finger of the right hand, while a negative response was represented corresponding left button to the index finger of the right hand. Participants were encouraged to proceed as quickly and accurately as possible. Accuracy and reaction times (RTs) (to the nearest millisecond) were recorded. Before the formal test, a short period of practice with a different set of sentences was provided.

### Data acquisition

MRI data were collected on a SIEMENS Trio 3.0 T scanner. Each subject lied on supine with the head in neutral position fixed comfortably by a belt and foam pads during the test. The scanning sessions included: (1) localizer, (2) T1 MPRAGE anatomy (176 sagittal slices, thickness/gap = 1.0/0 mm, in-plane resolution = 256 × 256, FOV (field of view) = 240 mm × 240 mm, TR (repetition time) = 1900 ms, TE (echo time) = 2.26 ms, flip angle = 15°), (3) EPI-BOLD (36 axial slices, echo-planar imaging pulse sequence, thickness/gap = 5.0/1 mm, in-plane resolution = 64 × 64, TR = 3000 ms, TE = 30 ms, flip angle = 90°, FOV = 240 mm × 240 mm). The sequence of scanning included a resting-state session, a task-state session, and another resting-state session. Only the first resting-state data was analyzed in this study. During the resting-state fMRI sessions, subjects were asked to remain as calm as possible and keep their eyes closed but not to fall asleep.

### Behavioral analysis

To examine changes in behavior performance over the course of the SD sessions, the mean RT and false rates (button pressed following a cue) about the accuracy of performance were computed respectively.

### MRI Data preprocessing

For the resting-state fMRI data, the first 2 volumes were discarded to avoid the possible effects of scanner instability and adaptation of subjects to the circumstances. The initial data preprocessing was performed using SPM8 (http://www.fil.ion.ucl.ac.uk/spm) and DPARSF 2.3 (http://www.restfmri.net/), including slice timing, head motion correction (a least squares approach and a 6 parameter spatial transformation), spatial normalization to the Montreal Neurological Institute (MNI) template (resampling voxel size = 3×3×3 mm^3^), and spatial smoothing (full width at half maximum = 6 mm Gaussian kernal). Further data preprocessing was performed using REST 1.8 (http://www.restfmri.net/) involved the removal of linear trends and filtering (0.01–0.08Hz) to reduce the influences of low-frequency drift and high-frequency noise.

For head motion correction session, we also calculated the frame-wise displacement (FD) which represents the scalar quantity of instantaneous head motion of each volume relative to its earlier neighboring volume. Recent studies have indicated that even little head motion can significantly influence the measures of resting-state fMRI [40,41,42,43]. In the present study, we take the following measures to reduce these motion effects: *i)* no subject was found with maximum displacement in one or more of the orthogonal directions > 0.5 mm or a maximum rotation > 0.5°; *ii)* the voxel-specific framewise displacement (FDvox) described as Yan et al.[42] was calculated for each subject, and considered as nuisance covariates in the statistical analyses.

### ALFF analysis

ALFF was calculated using REST 1.8 (http://www.restfmri.net/). Briefly, for the given voxel, the time series was first converted to the frequency domain using a Fast Fourier Transform. The square root of the power spectrum was computed and then averaged across a predefined frequency interval. This averaged square root was termed ALFF at given voxel. ALFF measures the absolute strength or intensity of low-frequency fluctuations.

In order to investigate alterations after 24h of sleep deprivation, we calculated ALFF at the typical frequency band (0.01–0.08 Hz), slow-4 (0.027–0.073 Hz) and slow-5 (0.01–0.027 Hz) band respectively. Then, ALFF of each voxel was computed for each participant and was further divided by global mean value to reduce the global effects of variability across participants [35,44].

### Statistical analyses

We first used paired-t tests to determine the effects of sleep deprivation on performance measures of reaction time (RT) and accuracy between SD and RW groups.

For ALFF, a one-sample one-sided t-test was performed within each group to determine whether the ALFF differed from the value of 1 [18,35], and a paired t test to see the differences between groups in the typical frequency band (0.01–0.08 Hz). For the comparison of frequency bands and their interaction between SD and RW groups, the data were analyzed with 2 × 2 within-subject repeated-measures ANOVA with condition (RW, SD) and frequency band (slow-4, slow-5) to minimize the chance of type I error. The regions that showed significant differences were then used for a post-hoc analysis. Voxels with a *p* value <0.01, cluster size >1053 mm^3^ (39 voxels), and corresponding to AlphaSim p<0.05 corrected (http://www.restfmri.net/) were considered to have a significant statistical difference between two groups.

### Brain–behavior relationships

To further evaluate the relationship between ALFF changes and task performance after SD, we examined the *Pearson's* correlation between ALFF values of peak voxels in group-difference areas and behavioral performance RT.

## Results

### Performance findings

In order to test the effects of sleep deprivation on the short-time memory, sixteen subjects performed semantic discrimination task before fMRI scanning (see methods) in RW and SD conditions. Behavioral measures of RT and accuracy for each condition were calculated (see methods). There was no significant difference in accuracy between the SD and RW groups. The SD group had significantly longer RT than that of the RW group (RW = 2,010.4 ± 227.17 (ms), SD = 2,275.1 ± 176.66 (ms), T = -3.858, d.f. = 15, P < 0.002).

### ALFF analyses in typical frequency band (0.01–0.08 Hz)

**Fig 1:**
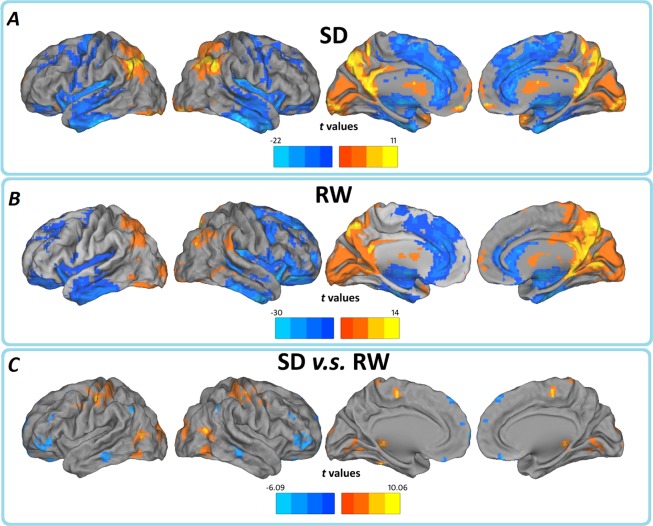
In typical frequency band (0.01–0.08Hz), regions of significant ALFF in the (A) SD and (B) RW groups separately, and their (C) between-group differences. The effects are significant at *p* < 0.05, FDR corrected; ≥100 contiguous voxels for one sample t test, and *p* < 0.05, AlphaSim corrected for paired t test. In the paired t test, cool color indicates that the SD group had decreased ALFF compared with the controls and the hot color indicates the opposite. Left in the figure indicates the left side of the brain.

Before comparing the between-group ALFF significances, we first report ALFF results from the typical frequency band (0.01–0.08 Hz) separately, both SD and RW groups showed a significant higher ALFF value level than global average in some regions including the visual cortex, posterior cingulate cortex (PCC)/ precuneus (PCu), bilateral thalamus, bilateral ventral medial prefrontal cortex (VMPFC), bilateral middle temporal gyrus (MTG) and dorsolateral prefrontal cortex (DLPFC), mainly along the midline and visual cortices. We note that many regions are components of what is known as the most prominent intrinsically connected hubs, namely the DMN. This finding is consistent with the conclusion that these regions represent the most prominent and functional core underlying resting brain dynamics [24]. Obviously the ALFF strength was lower in SD group, comparing with the RW control, the results may indicate a global change of spontaneous brain activity pattern (Fig. 1 and S1 Fig.).

We then contrasted these ALFF patterns between the two groups, thereby identifying the consequential differences resulting from 24h SD, relative to a normal night’s sleep. As expected, we find significant ALFF decreases in the bilateral OFC (BA 11/47), bilateral DLPFC (BA 46), and right inferior parietal lobule (IPL, BA39/40) following 24h SD. Interestingly, we also observed that the SD group showed increased ALFF in the left somatosensory cortex (SMC), left fusiform gyrus (BA 37) and visual cortex (BA 18/19) (Fig. 1 and S1 Fig.; Table 1), few similar results were reported previously. It is unclear whether such increase simply imply a rise overall variance in the ambient BOLD activity (reflective of higher metabolic activity) or a reduced LFF activity in the SD. To clarify this issue, we further measured the regional LFF changes by examining regional coherence in a pure resting state. Regional coherence was assessed using regional homogeneity (ReHo) method [45]. Briefly, ReHo measures the temporal synchronization of the time series of nearest neighbors (for detail, see [45]), using this procedure, we found that the SD group also showed significantly increased ReHo in the SMC and visual cortex, indicated SMC and visual cortex in this compensation.

**Table 1:** In the typical frequency band, detailed information for clusters showing group ALFF differences at the given threshold (*p* < 0.05, AlphaSim corrected).

**Brain regions**	**MNI coordinates**			**BA**	**L/R**	**Voxels**	**T values**
	x	y	z				
Medial Frontal Gyrus	15	66	−3	11/10	R	54	−5.2249
Superior Frontal Gyrus	−42	48	0	10/11/46	L	91	−4.6173
Middle Frontal Gyrus	−51	33	27	8/9/46	L	61	−5.0072
Inferior Frontal Gyrus	−51	36	0	10/45/46/47	L	62	−4.6245
Inferior Frontal Gyrus	51	15	0	22/45/47	R	141	−4.9034
Inferior Parietal Lobule	66	−48	24	39/40	R	156	−6.0926
Precentral Gyrus	36	−24	36	2/3/4/6	R	319	6.5118
Precentral Gyrus	−36	−12	57	1/2/3/4/6	L	652	10.0644
Middle Occipital Gyrus	−39	−87	0	18/19	L	413	6.7853

### ALFF changes in different frequency bands

Previous studies used EEG or MEG showed sleep deprivation induced frequency-specific changes of brain networks [31,32,33], some recent studies indicate different frequency bands between low-frequency (i.e. 0.01–0.08 Hz) may be distinct in several brain regions [24]. In order to investigate specific alterations after one night of SD, we also subdivided the low frequency range into four bands as previously defined [24,27]: slow-5 (0.01–0.027 Hz) and slow-4 (0.027–0.073 Hz). To test for the presence of regional differences in ALFF at two bands, we carried out two-way repeated measures ANOVA analysis between the slow-5 and slow-4 bands for ALFF.

**Fig 2:**
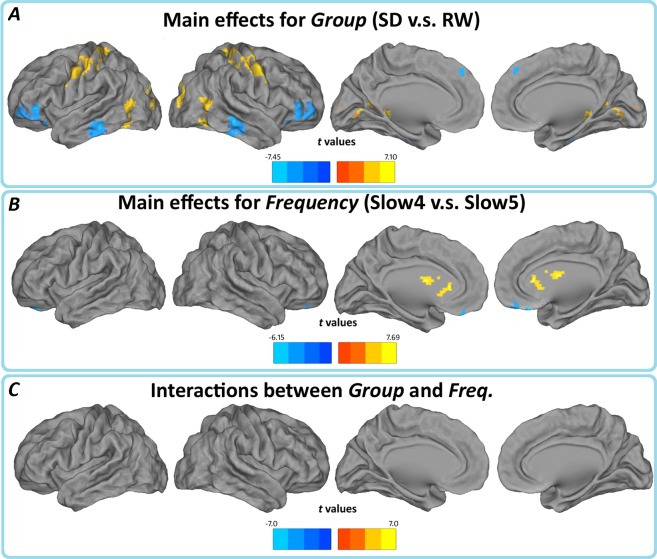
(A) Main effects for group, (B) frequency band, and (C) their interactions. Hot color represents higher ALFF in the SD group (Slow-4) than in the control group (Slow-5), whereas cool color represents lower ALFF. The results were obtained by a 2×2 repeated-measure ANOVA.

Main effects from the two-way repeated measures ANOVA are shown in Fig. 2. Brain regions with a main effect of group, including bilateral orbitofrontal cortex (OFC), cerebellar tonsil, right inferior temporal gyrus (rITG), bilateral fusiform gyrus (FG), right parahippocampal gyrus (rPHG), right middle frontal gyrus (rMFG), left superior frontal gyrus, left inferior temporal gyrus right angular gyrus, right middle cingulate gyrus (SD<RW); bilateral fusiform gyrus (BA 19/37), left inferior occipital lobe(BA 18), bilateral lingual gyrus(BA 18/30), left middle temporal gyrus (BA19/37/39), bilateral postcentral gyrus and precentral gyrus (BA 1/2/3 /4/6/40/43), and right paracentral lobe (BA 6) (SD>RW).

**Fig 3:**
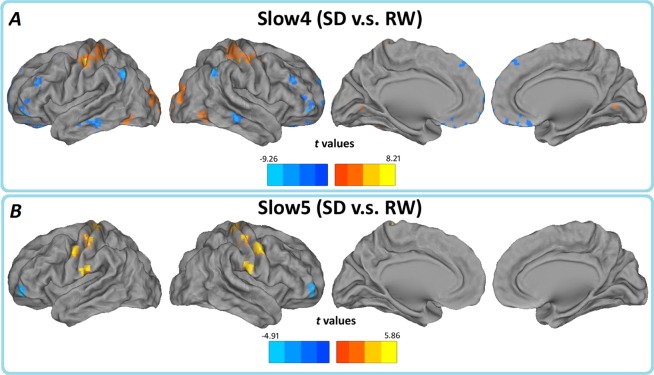
Paired t test for the (A) Slow-4 and (B) Slow-5 frequency band, *p* < 0.05, AlphaSim corrected. Cool color indicates that the SD group had decreased ALFF compared with the controls and the hot color indicates the opposite. Left in the figure indicates the left side of the brain.

Brain regions showing a significant main effect for frequency band were identified in the bilateral orbitofrontal cortex, right fusiform gyrus (BA 38), (BA 11/47), right temporal gyrus(BA 21) (Slow-4<Slow-5); left cerebellum, left brainstem, bilateral hippocampus (BA 13/47), bilateral thalamus, and caudate (basal ganglia) (Slow-4>Slow-5). There were no significant interactions between frequency band and different groups (Fig. 2). Further post-hoc t test reveals that the group differences in ALFF in the slow-4 band were greater than those in the slow-5 (Fig. 3, Table 2 and 3).

**Table 2:** In the Slow-4 (0.027–0.073Hz), group ALFF differences at the given threshold (*p* < 0.05, AlphaSim corrected) were shown.

**Brain regions**	**MNI coordinates**			**BA**	**L/R**	**Voxels**	**T values**
	x	y	z				
Superior Frontal Gyrus	−9	48	51	6/8	L	257	−5.5499
Superior Frontal Gyrus	−15	60	21	9/10	L	130	−5.0127
Medial Frontal Gyrus	15	48	−24	11	R	69	−5.5202
Middle Frontal Gyrus	−48	36	27	9/46	L	90	−5.0586
Inferior Frontal Gyrus	−27	45	−15	11/47	L	90	−5.1374
Inferior Frontal Gyrus	51	15	3	13/38/22/45/47	R	209	−5.6583
Lingual Gyrus	21	−90	−3	17/18	L	111	4.1698
Angular Gyrus	42	−57	33	40	R	92	−5.137
Inferior Parietal Lobule	−54	−63	42	39/40	L	74	−9.2636
Postcentral Gyrus	−39	−21	51	2/3/4/6/40	L	504	8.2133
Middle Occipital Gyrus	−15	−93	9	18/19	L	204	4.0821
Superior Frontal Gyrus	−9	24	−15	11/47	L	351	−6.8423
Middle Frontal Gyrus	−42	42	−6	10/11	L	92	−3.984
Thalamus	21	−15	12	-	R	55	4.3722

**Table 3:** In the Slow-5 (0.01–0.027 Hz), group ALFF differences at the given threshold (*p* < 0.05, AlphaSim corrected) were shown.

**Brain regions**	**MNI coordinates**			**BA**	**L/R**	**Voxels**	**T values**
	x	y	z				
Middle Frontal Gyrus	−39	51	-6	10/11	L	100	−4.9078
Postcentral Gyrus	−57	−21	21	3/4/6	L	395	5.8595

**BA**, Brodmann's area; **MNI**, Montreal Neurological Institute; x, y, z, coordinates of primary peak locations in the space of MNI; t, statistical value of peak voxel; *p*<0.01, corrected for AlphaSim multiple comparisons.

### Correlations between ALFF and behavior variables in the SD group

**Fig 4:**
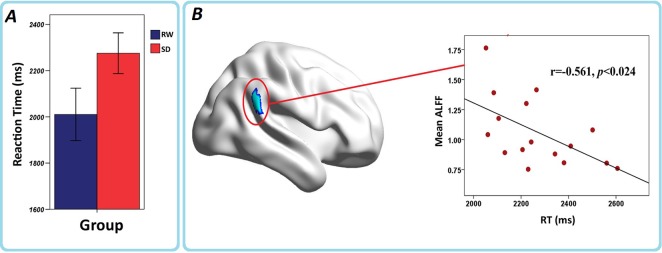
(A) Performance RTs for the RW (blue) and SD (red) sessions, and (B) the correlation between RTs and ALFF change in the right IPL in sleep deprivation group. The greater the prolongations in RT from rest to sleep deprivation, the greater the decrease in ALFF in right IPL. The results were visualized with the BrainNet Viewer (http://www.nitrc.org/projects/bnv/).

To evaluate the relationship between ALFF and behavior variables in the SD group, we found that negative correlations of behavior performance and ALFF patterns appeared mainly in the right IPL across the typical frequency band (Fig. 4).

## Discussion

We investigated the frequency-dependent effects of sleep deprivation on low frequency oscillations of BOLD signals measured by resting-state fMRI. 24 h sleep deprivation caused the decrease of ALFF in the bilateral OFC, bilateral DLPFC, and right IPL areas while the increase of ALFF in the left SMC, visual cortex and left FG areas. These changes were much more significant in Slow-4 frequency band. Behaviorally, 24h sleep deprivation significantly affected reaction time in the semantic task, and this change was also negatively correlated with decreased ALFF in the right IPL. These findings may offer SD-related frequency-dependent alterations in amplitude patterns and provide novel insights into understanding how ongoing spontaneous brain activity respond and compensate to prolonged wakefulness.

Electroencephalography (EEG) studies have found that sleep deprivation are associated with tonic decreases in EEG amplitude of spectral features associated with alertness [46,47,48], and also frequency-specific changes in brain networks [31]. Although there is still lack of consensus about the significances of different frequency bands (e.g. slow-4 and slow-5 frequency band) in BOLD signals, previous studies have indicated that amplitudes in the slow-4 were higher in the basal ganglia, thalamus and several sensorimotor regions while lower within the ventromedial regions [49]. We observed more significant changes of ALFF in the slow-4 band than that of in the typical frequency band, especially in the OFC, IPL, and VMPFC in a sleep-deprived brain. This can be interpreted that slow-4 band could be more sensitive in detecting changes of spontaneous brain activity in the OFC, IPL, and VMPFC for sleep-deprived brain. Previous study did not find the significant alternations of the DMN in the spectral power between the (0–0.1 Hz) and (0.1–0.25 Hz) frequency in the sleep-deprived brain [15]. One of main cause was derived from partial sleep deprivation by allowing 3.5 hours sleep, while the present study had much longer 24 hours sleep deprivation. On the other hand, they only focused on the DMN while we used the whole brain ALFF analysis.

Our results confirmed the functional alterations of resting state networks (RSNs) after sleep deprivation in previous studies [12,13,15]. It has been well established that intrinsic brain activity detectable by resting-state fMRI is organized into spatially distinct resting state networks (RSNs). Mapping these intrinsic connections constitutes a major goal of recent efforts to the human functional connectome [50]. Among these spontaneously synchronized neuronal networks, the “task-negative” (DMN) and its anticorrelated “task-positive” networks (ACN) [51,52] have received most attentions. One recent study found that insomniacs have increased insula coactivations within brain networks associated with salience and arousal, and may, in part, be explained as that chronic sleep loss put a burden on brain networks coordinating. Since a primary role of the salience network is proposed to enable switching between the DMN and ACN[53]. This dysfunctional integration within and between DMN-ACN is consistent with our findings. The DLPFC recruited the working memory tasks [54,55,56,57]. It becomes one of the “task-positive” regions (ACN) and has been implicated to be responsible for the failure in working memory for the sleep-deprived brain [51,54,55,56,57]. One recent study reported a reduced anti-correlation between the DMN and ACN nodes at both task-related and resting states [13]. An early PET study also found a global CMRGlu decline and a relative decrease in these regions [58]. Despite lack of statistically significant correlations among behavior variables, the relatively decreased ALFF in the DLPFC after sleep deprivation may represent a decline in memory encoding, though it represents a recoverable process.

Our study also showed the decrease of ALFF signals in the bilateral OFC (BA 11/47) and right inferior parietal lobule (IPL, BA39/40) following 24h of sleep deprivation. These regions are main cores of the DMN [18] characterized by more energetic metabolic and neural activity at rest. These areas have been suggested as being engaged in internal spontaneous cognitive activity, including autobiographical memory retrieval and envisioning the future when individuals are not focusing on the external environment [18,51,59,60]. Alterations of these regions may be associated with the process of energy metabolism and cognitive operations after SD. A proposed function for sleep is brain energy restoration [61], and several studies find increased anabolic processes to the restorative biosynthetic courses occurring during sleep [62]. Sleep deprivation could be energetically costly due to increased energy expenditure for maintaining wakefulness. Regional hypometabolism in the prefrontal and parietal cortex often occurred after sleep deprivation [58,63,64]. The SD reduced not only resting metabolism but also functional connectivity in the VMPFC with the amygdala and other main cores of DMN [12,13,15,21]. The decreased volumes of gray matter in the OFC have been reported in patients with chronic sleep disorder [65,66] due to chronic sleep loss. The OFC, known as the limbic system, is thought to be responsible for mediating the interactions between emotional processes and cognitive functions [67,68]. This area is particularly vulnerable to sleep deprivation [69]. Damages to the OFC have similar effects that result from the sleep deprivation on decision making, though it is less severe [8,70,71]. It is also well established that SD has a therapeutic effect on depression [16,72]. Negative cognitive biases in depression are facilitated by increased influence from subcortical emotion processing regions combined with attenuated top-bottom cognitive controls [72]. Studies about depression has found increased functional connectivity between the VMPFC and other cores of DMN and increased ALFF in the frontal cortex [73]. However, our data did not reveal a significant correlation between the ALFF values in the OFC and the RT values in the SD group (p = 0.73). This may need more specific cognitive performances. The decreased regional activity in the OFC seems to be the most consistent alteration after one night of SD and represents maladaptive emotion regulation [12,68]. It has been found that functional changes in the parietal cortical regions may be the most reliable neural features of an individual’s response to sleep deprivation [2,13]. The changes of IPL within the DMN are consistently impaired and may represent an early marker for the effects of sleep deprivation [13]. Our study also found that the decreased ALFF in the IPL was frequency-dependent—low ALFF on the right side across the typical frequency band (0.01–0.08Hz) in contrast to much higher ALFF on the both side (especially in the left side) across the slow-4 frequency band. Moreover, there is a significant negative linear correlation between the ALFF changes in the right IPL and reaction time for cognitive performance, and it was notioned that activity changes in the IPL may reflect an imaging marker for sleep deprivation. Together, the decreased ALFF in these key DMN regions may be dependent on prior sleep, sleep deprivation may disrupt the amplitude patterns of brain oscillatory activity, offer novel insights into spontaneous brain activity associated with sleep loss both cognitively and emotionally.

One interesting finding was the increased ALFF in the left SMC, visual cortex and left FG after sleep deprivation. It is unclear whether such increase simply imply an increased LFF activity or a rise overall variance in the ambient BOLD activity (reflective of higher metabolic activity) in the SD. To clarify this issue, we further measured the regional LFF changes by examining regional coherence in the typical frequency band. Regional coherence was assessed using regional homogeneity (ReHo) method [45]. Briefly, ReHo measures the temporal synchronization of the time series of nearest neighbors (for detail, see [45]). A paired-t test was carried out on group ReHo map in a voxel-wise way. We found that the SD group also showed significantly increased ReHo in the SMC. Using a series of addition/subtraction tasks, previous PET study find that sleep deprivation changed the waking cerebral metabolic rate for glucose (CMRGlu) with the regional relative increases in the visual cortex [63], left SMC, lateral superior occipital cortices, lingual and FG [64]. These changes were much greater after 48 and 72 h than that of after 24 h SD [58]. Furthermore, these regions are consistent with previous findings of increased activation in sleep deprivation during the performance of cognitive task [74]. They proposed that the increased activations implicated the brain’s exertion of voluntary control to remain awake and performance [74]. The FG and visual cortex are involved in memory process [74], and we speculated that increases in these regions may reflect the compensatory recruitment though this conclusion needs further clarifications. The recruitment of increased brain regions may reflect an attempt to sustain alertness and cognitive performance despite a continuing decline in the prefrontal-parietal activity.

## Limitations

There are several limitations in this study. Firstly, a relatively small sample, the statistical power lowers and limited, so the results can hardly survive a strict multiple comparison correction (e.g. FDR or FWE correction). Future studies could use a larger sample size to increase the statistical power of the study. Secondly, lack of objective assessment of sleep quality and daytime sleepiness. Although none of our participants had self-reported sleep disorder, an elusive impact of sleep habits on intrinsic brain activity cannot be excluded. However, the impact would be very limited.

## Conclusion

Using ALFF as an index of regional intrinsic brain activity in resting-state, we find that 24h sleep deprivation leads to altered regional activity in the DMN and ACN. The decreased ALFF emerged mainly in the OFC, DLPFC and IPL while increased ALFF in the left FG, visual cortex and SMC. These regions are consistent with previous findings from cognitive tasks explained in terms of a cognitive impairment, maladaptive emotion regulation and compensatory recruitment hypothesis. Further, the alterations of ALFF are more significant in the Slow-4 frequency band in contrast to the typical frequency band. Our results highlighted sleep deprivation can induce frequency-dependent brain oscillatory activity changes. These findings may provide insights into the understanding of the maladaptive process of sleep deprivation and of how ongoing spontaneous brain activity respond and compensate to sleep deprivation.

## Supporting Information

S1 Fig Main effects for group and frequency band. Hot color represents higher ALFF in the SD group (Slow-4) than in the control group (Slow-5), whereas cool color represents lower ALFF. The results were obtained by a 2×2 repeated-measure ANOVA.
(TIF)

S2 Fig Paired t test for the Typical, Slow-4 and Slow-5 frequency band, p < 0.05, AlphaSim corrected. Cool color indicates that the SD group had decreased ALFF compared with the controls and the hot color indicates the opposite. Left in the figure indicates the left side of the brain.
(TIF)
