# Supplementary figures and images for: Frequency-Dependent Changes of Local Resting Oscillations in Sleep-Deprived Brain
Source: PLoS One. 2015 Mar 23;10(3):e0120323. doi: 10.1371/journal.pone.0120323 (PMC4370559; doi:10.1371/journal.pone.0120323)

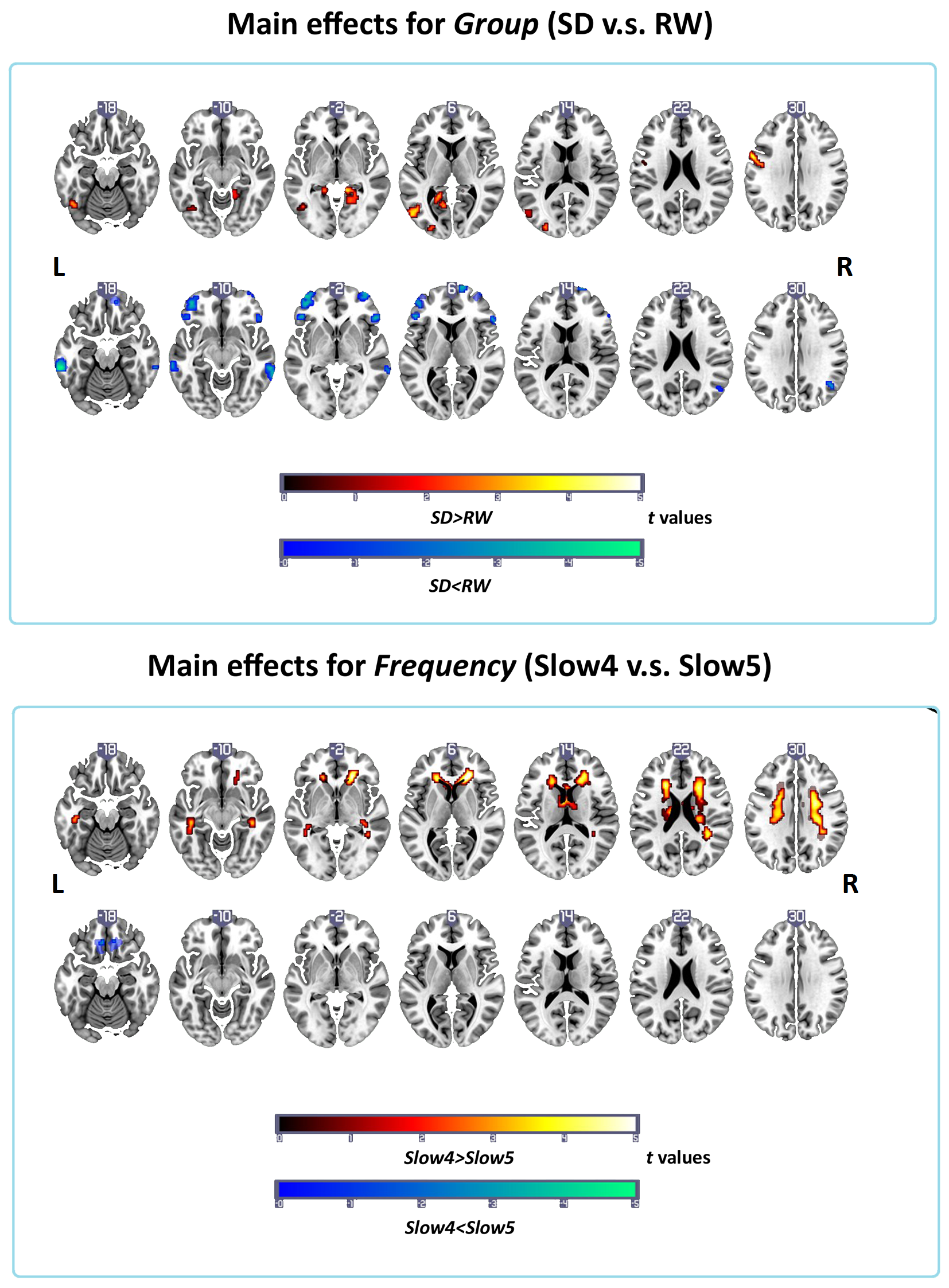

Supplement: S1 Fig — Hot color represents higher ALFF in the SD group (Slow-4) than in the control group (Slow-5), whereas cool color represents lower ALFF. The results were obtained by a 2×2 repeated-measure ANOVA. (TIF) [file pone.0120323.s001.tif]

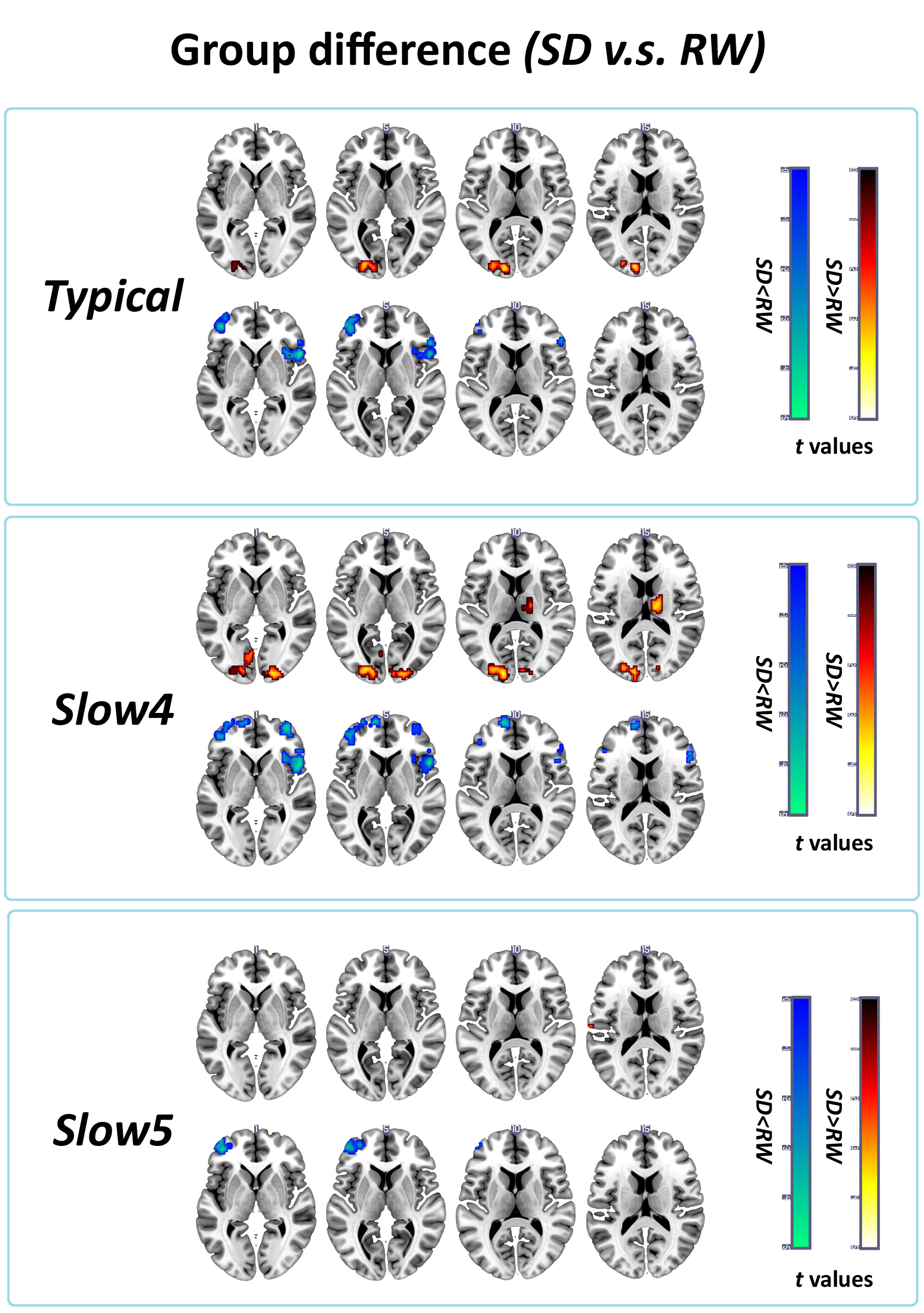

Supplement: S2 Fig — Cool color indicates that the SD group had decreased ALFF compared with the controls and the hot color indicates the opposite. Left in the figure indicates the left side of the brain. (TIF) [file pone.0120323.s002.tif]
